# Towards Better Pharmaceutical Provision in Europe—Who Decides the Future?

**DOI:** 10.3390/healthcare10081594

**Published:** 2022-08-22

**Authors:** Denis Horgan, Tanja Spanic, Kathi Apostolidis, Giuseppe Curigliano, Joanna Chorostowska-Wynimko, Hans-Peter Dauben, Jonathan A. Lal, Rafal Dziadziuszko, Christine Mayer-Nicolai, Marta Kozaric, Bengt Jönsson, Iñaki Gutierrez-Ibarluzea, Marie-Helene Fandel, Ruth Lopert

**Affiliations:** 1 European Alliance for Personalised Medicine, 1040 Brussels, Belgium; 2 Department of Molecular and Cellular Engineering, Jacob Institute of Biotechnology and Bioengineering, Faculty of Engineering and Technology, Sam Higginbottom University of Agriculture, Technology and Sciences, Prayagraj 211007, India; 3 Europa Donna, The European Breast Cancer Coalition, 20149 Milan, Italy; 4 European Cancer Patient Coalition, 1210 Brussels, Belgium; 5 Department of Oncology and Hemato-Oncology, University of Milan, 20122 Milan, Italy; 6 European Institute of Oncology, IRCCS, 20139 Milan, Italy; 7 Department of Genetics and Clinical Immunology, National Institute of Tuberculosis and Lung Diseases, 26 Plocka Str., 01-138 Warsaw, Poland; 8 EuroScan International Network e.V., 50825 Cologne, Germany; 9 Institute for Public Health Genomics, Department of Genetics and Cell Biology, GROW School of Oncology and Developmental Biology, Faculty of Health, Medicine and Life Sciences, Maastricht University, P.O. Box 616, 6211 LK Maastricht, The Netherlands; 10 Department of Oncology and Radiotherapy of the Medical University of Gdansk, 80-214 Gdansk, Poland; 11 Global Regulatory and Scientific Policy (GRASP), Merck, 64293 Darmstadt, Germany; 12 Department of Economics, Stockholm School of Economics (SSE), P.O. Box 6501, 113 83 Stockholm, Sweden; 13 Department of Knowledge Management and Evaluation of the Basque Foundation for Health Innovation and Research (BIOEF), 48902 Barakaldo, Spain; 14 Corporate Public Affairs, Sanofi, 1831 Diegem, Belgium; 15 Organisation for Economic Co-Operation and Development, OECD, 75775 Paris, France

**Keywords:** pharmaceutical legislation, personalized medicine, innovation, unmet medical need, access, security of supply, efficiency, policy framework, regulation

## Abstract

Significant progress has been achieved in human health in the European Union in recent years. New medicines, vaccines, and treatments have been developed to tackle some of the leading causes of disease and life-threatening illnesses. It is clear that investment in research and development (R&D) for innovative medicines and treatments is essential for making progress in preventing and treating diseases. Ahead of the legislative process, which should begin by the end of 2022, discussions focus on how Europe can best promote the huge potential benefits of new science and technology within the regulatory framework. The challenges in European healthcare were spelled out by the panellists at the roundtable organised by European Alliance for Personalised Medicine (EAPM). Outcomes from panellists’ discussions have been summarized and re-arranged in this paper under five headings: innovation, unmet medical need, access, security of supply, adapting to progress, and efficiency. Some of the conclusions that emerged from the panel are a call for a better overall holistic vision of the future of pharmaceuticals and health in Europe and a collaborative effort among all stakeholders, seeing the delivery of medicines as part of a broader picture of healthcare.

## 1. Introduction

The evolution of healthcare has a history as long as human beings—and has been a matter of inquiry and contention for almost as long. The appearance of science/research-based medicines is a more recent story, but the potential that pharmaceutical therapies hold for maintaining and improving healthcare and quality of life has made discussion of the subject all the more intense [1,2,3]. In Europe, the seat of so much innovation in this field in modern times, it is taken for granted that pharmaceutical development should be influenced by public policy as much as by pioneering philanthropy or private profit. However, that leaves open the question of how much influence each should exert—and the question has become explicit in the plan for the European Union to review its decades-old legislation governing pharmaceuticals. In the run-up to the legislative process that is scheduled to begin by the end of 2022, discussions are focusing on how Europe can best promote the huge potential benefits of new science and technology within a regulatory framework that, according to the European Commission’s 2019 ‘pharmaceutical strategy’, envisages protecting both the interests of Europe’s patients and the sustainability of the member states’ public health systems—a dual objective that is itself not without controversy, as detailed below. The questions are multiple: What frameworks can foster innovation, including in areas of unmet medical need (UMN)? What mechanisms can help ensure access to medicines? How can security of supply be improved? How should public policy adapt to new scientific and technological developments? Furthermore, how to bring greater efficiency into healthcare provision? The answers are more elusive. There is wide agreement on the objective of better, more equitable, more reliable, and more efficient healthcare [4]. However, at the same time there are sharply differing shades of opinion on how this should be done. Furthermore, the discussions are now conditioned by stark new circumstances, including COVID-19’s demonstration of the continuing vulnerability of global populations to pandemic infection, and the Ukraine conflict’s demonstration of the fragility of the global political order—and the concomitant and consequent disruption these events have imposed on health services and health. As a widely based healthcare organisation dedicated to preparing for the future, European Alliance for Personalised Medicine (EAPM) hosted a day-long series of panel discussions in March among representatives of key stakeholder groups to ventilate these questions. Public health decision makers, representatives from the European Commission, Members of the Parliament, patient organisations, Health Technology Assessment (HTA) representatives, and umbrella organisations representing interest groups and associations actively engaged in the field explored the common challenges and common frameworks to best support improving the quality of life of citizens across Europe, with the best possible alignment of national and EU level approaches to healthcare provision. This paper provides an overview of the discussions, highlighting the questions that are still open as well as the areas of consensus, including some of the tentative pathways that were suggested for further exploration in search of solutions.

It is no coincidence that the agenda has an echo of the European Commission’s public consultations on its plans for legislative review, which focused on UMNs, incentives, unequal access, responding to innovation, the burden of regulatory procedures, and shortages [5]. The European Commission’s major overhaul of rules that govern medicines—the revision of the general pharmaceutical legislation—is tentatively set to be published in December 2021 [6]. A proposal to update a Council recommendation on cancer screening is also scheduled for September 2021 [7]. 

Neither is it a surprise that the liveliest issues raised during the meeting coincide with many of the public comments submitted to the Commission’s consultations as it prepares its upcoming proposals. The dramatic improvement in the health of the citizens of Europe over the last two centuries has transformed the continent and the lives of the people living in it. The literature review explored whether Europe is able to seize the new benefits that science, technology, and forward-thinking public-policy decisions could confer on current and future generations of Europeans—or is it losing the will and capacity to grasp the fruits of progress? With the upcoming revision of EU pharmaceuticals legislation, and drawing lessons from the COVID-19 pandemic, the EAPM organised a consensus panel to examine challenges and opportunities from this new legislation. For example, in the framework of UMN, it takes on average more than a decade to develop a new medicine. The journey is fraught with setbacks, false horizons, and very limited certainty about whether a research asset or treatment will effectively address an UMN. Similarly, when research started on mRNA technology, few would have imagined it would play a central role in fighting a pandemic. The Commission is now looking at creating a list of therapeutic areas recognized as representing an UMN in the EU.

How can such a list help in averting health threats that we are not aware of today? How can patients’ perspectives, preferences, and insights be integrated into such a list? Is the EU creating standards that will limit regulatory incentives only to therapeutic areas that feature on a list? This is unlikely to ease the current relative decline in EU global competitiveness, which is one of the stated goals of the EU pharmaceutical strategy. On the most recent figures, 20% of innovative medicines come from the EU, compared to 50% from the US—a reversal of the situation of 25 years ago. The EU has fallen far behind, while competition from Asia and in particular China is growing. European patients and Europe’s economy suffer accordingly. So as the Commission is poised to make the innovation process in the EU even more challenging, and possibly less rewarding, for pharmaceutical developers to file their products in the EU, it is appropriate to reflect on measures that could better ensure an EU regulatory system that remains attractive for the development of innovative treatments addressing all types of UMNs.

The challenges in European healthcare were clearly spelled out by the panellists at the EAPM workshop: wide inequalities across Europe in access, particularly to new medicines; persistent lack of effective treatment for many conditions, particularly rare diseases; growing strains on the financial sustainability of healthcare systems, resulting in part from newer and often more expensive treatments; delays in adoption or availability of innovative treatments; and regulatory systems no longer in tune with contemporary science and technology, or responsive to new opportunities in healthcare. Highlights from the diverse day-long discussions have been summarised to stimulate further discussion and re-arranged in this paper under five headings: innovation, UMN, access, security of supply, adapting to progress, and efficiency.

## 2. Materials and Methods

**Figure 1:**
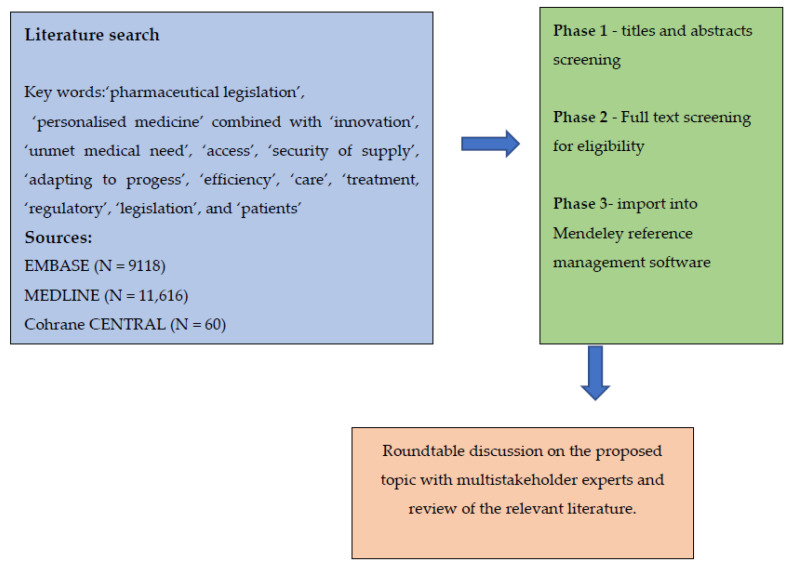
Applied research strategy; after literature search of relevant articles, an expert panel was organised to discuss the proposed topic and literature findings.

Inputs presented in this paper are based on a systematic literature search undertaken to develop an overview to contextualise challenges and opportunities related to the plan for revised pharmaceutical legislation. This was complemented by a multistakeholder expert workshop organised by the EAPM earlier in 2022. A narrative literature search was performed in EMBASE (via Ovid), MEDLINE (via PubMed), and Cochrane CENTRAL. Records identified through the designed search strategy were imported into Mendeley reference management software. Search terms were based on terminology used by EAPM and the authors’ own experiences in the field and focused on: ‘pharmaceutical legislation’ and ‘personalised medicine’ combined with ‘innovation’, ‘unmet medical need’, ‘access’, ‘security of supply’, ‘adapting to progress’, ‘efficiency’, ‘care’, ‘treatment, ‘regulatory’, ‘legislation’, and ‘patients’ (Figure 1). Only articles written in English, published after 2005, and full texts were included in the review. Panel members were drawn from key stakeholders whose interaction created a cross-sectoral, relevant, and dynamic discussion forum. These participants included public health decision makers, representatives from the European Commission, Members of the Parliament, patient organisations, and umbrella organisations representing interest groups and associations engaged in the field. Experts discussed all topics (innovation, unmet medical need, access, security of supply, adapting to progress, and efficiency) together but were also allocated to groups based on their individual expertise. The meeting discussed the literature findings from the perspective of different stakeholders and identified potential pitfalls and advantages in the current Commission proposals. The expected outcome of the expert panel was to feed into activity that will support policy engagement at the EU level and national level related to fostering innovation, including in areas of UMN, ensuring access to affordable medicines, improving security of supply, adapting to new scientific and technological developments, and reducing red tape.

## 3. Discussion: Different Challenges and Opportunities

Overall, the most widely supported sentiment to emerge from the panellists was a call for a better sustainable holistic vision of the future of bringing pharmaceuticals, early diagnosis and health into Europe’s healthcare system. This, it was concluded, requires a more determined collaborative effort among all stakeholders to perceive the delivery of medicines as part of a broader picture of healthcare. The optimum approach is to replace the current common silo thinking with a consensus-based vision of common interest in a constantly improving and adapting service to Europe as a whole, knowing that health is wealth and vice versa, which takes account of the following.

### 3.1. Innovation

The right to innovation is enshrined in the charter of patients’ rights in some EU member states, but gaps in innovation—both in the development of new diagnostics and therapeutics, and in their availability even when authorized—were repeatedly reported by representatives of clinicians, medical societies, and patients, particularly in the respiratory field, neurology, lung cancer, and paediatric oncology [4,8]. Over the last two decades, the pathways to innovation have changed significantly, and so have the actors: patients and citizens are now more actively involved, and the utilisation of data has dramatically advanced. There are evident expectations among patient groups of innovations promised and still to come, in diagnosis, in medicine, in devices, and in crossovers between medicines and medical devices [9]. The European Commission depicts the prospect of revised legislation as “an exciting moment” for innovation, and candidly recognises that this is an area where Europe is underperforming and potentially losing competitiveness, because despite major technological developments, there are gaps in how Europe is able to accommodate and exploit them [10,11]. The industry repeatedly points to figures suggesting that Europe is losing its edge in terms of innovative companies and activity as it is matched and overtaken by competitors in the USA and the Far East [12].

At issue are questions for medicine developers (and their investors) of the attractiveness of filing in the EU—conditions judged in the light of policies such as regulatory incentives, agile assessment frameworks, data protection, and market exclusivity [13]. However, for health authorities and payers, as well as many academics and patient representatives, the questions often relate more to judgements of the quality and extent of evidence in support of applications for approval, price, return on public investment, or data privacy [14,15]. HTA and payer bodies frequently complain that supporting data for applications are insufficient for them to reach an informed conclusion on a product’s merits [16].

The enthusiasm for innovation is widely shared. The examples of rapid and remarkable innovation during the pandemic have acquired something of a legendary quality—ranging from the Mercedes FI involvement in producing a ventilation device [17] to the capacity to design a DNA or mRNA vaccine [18] within days of acquiring the genetic sequence of a new pathogen, to identify a lead candidate for clinical trials within weeks and have millions of doses manufactured within months [19].

However, when the current incentives regime is challenged, as it frequently and loudly is in the current pre-legislative discussions, drug developers question the commitment of the EU to maintaining support for innovation [20]. The same sense of challenge is felt when definitions of the “right sort” of innovation arise, prompting fears that incremental smaller improvements may be excluded from rewards. Disquiet is provoked by dismissals of precision medicine as a tactic merely to run expensive tests to justify giving an even more expensive drug—when it may also be depicted as allowing optimised outcomes by better use of available resources at the points where they have most benefit, to the advantage of all (patients, healthcare systems and society) [21,22].

Staunch defenders of innovation argue that it doesn’t emerge from nowhere. They insist it is the result of research, private or public, and of the related investment. In other words, patients get what has been invested in. Investment is the precondition to a conducive framework, and investment depends on a climate with a degree of certainty and predictability. Studies presented suggested that industry is increasing its investments in cancer research much faster than the public sector [23]. A conducive framework for innovation is seen by some as providing regulatory or infrastructure support (dealt with below under “efficiency”), but by others as encouragement in terms of economic returns, as incentives for research or in terms of pricing [24,25]. 

However, there is caution perceptible among many stakeholders over unconditional endorsement of the merits of innovation. Some patient groups suggest that patients can feel alienated by the rapidly changing cancer landscape. Despite the increased chances of cure arising from innovation, some patients have difficulty in grasping the concept of personalised medicine, and many feel uninvolved in decisions about their care [26,27]. Defenders of personalised medicine counter that lack of awareness is often due to insufficient testing, since efficient and broad testing such as Next -Generation Sequencing (NGS) is not reimbursed.

Even among those who espouse innovation, there are many who insist it must be of significant scale to justify attention: this manifests itself in a reserved attitude to incremental innovation, sometimes depicted as a disservice to patients and as a mechanism for industry to claim unjustified support [28].The Commission concedes that incremental innovation “can as well be something good”, but insists that the focus—and thus the highest level of incentives—should be on innovation for unmet medical needs—which opens up another area of considerable divergence of views (see below, “unmet medical need”) [5]. Luxembourg, as a small country, claims to follow an approach which is pragmatic and flexible, accepting incremental innovation even for something relatively simple but that provides ease to the patient or the healthcare provider, such as vaccines that do not need deep-cold storage, or inhalers that are easier to use and improve compliance, or a methodological shift to a more comprehensive approach to a disease—including prevention and vaccination.

Technology developers point to an intrinsic difficulty in dividing innovation into breakthrough or incremental, suggesting an underlying misconception. Innovation, it is argued, is a continuum, albeit not a linear one, that defies such a binary categorisation. At initiation, it is impossible to predict how big a step a study will lead to: it may produce innovation that is a quantum leap, but it might be just a smaller step upwards. In this logic, developers naturally aim for the best but must go where the science permits [29]. Or as another technology developer put it, “We know by experience that innovation comes in increments. Only rarely do we see quantum leap improvements.” The breakthroughs represented by proton pump inhibitors, or by direct acting antivirals on hepatitis C, have to be set against the many small incremental changes made to both beta blockers and insulin, or in creating more effective cancer cocktails [30,31,32]. Without reward for those incremental smaller improvements over the first product, the better treatments available today might not have been developed, the argument runs. The pharmaceutical industry acknowledges that an innovation can bring different levels of added value to patients and society, but it emphasises that any added-value should be recognized and brought to the patients, because skipping the incremental path of innovation would prevent many patients from improving their condition, and also eliminate many candidate drugs that may lead to breakthrough innovations [33].

In the view of the Commission, there should be what it suggests could be a more balanced system of regulatory incentives, in which rewards for innovation are tied to conditionalities so as to better address issues of access [34].Innovation is linked by others inextricably to creating “a sustainable biomedical innovation system” in which novel medical products are treated as global public goods—subordinating the concept of return on investment to the concept of ease of access (on which, see below, “Access”) [35]. Similarly, World Health Organization (WHO) Europe talks of supporting innovation in “partnerships”. Meanwhile, buyers are calling for “meaningful needs-driven innovation focused on patients and on public health”. They speak of “very high prices for some products and weak evidence” and call for more and better data as early as possible for approving new products, with more transparency in the system and equitable access for patients. 

However, while incentives—particularly for innovation for rare diseases and paediatric treatments—have been heavily and critically scrutinised in some quarters, and appear to be in line for some pruning under the Commission’s proposals, drug developers argue that without incentives, the generation of potential treatments for smaller populations (and archetypically in the case of rare diseases) is less likely in view of the high research and development costs [36]. The current incentive mechanisms have, it is argued, shown their efficacy with a large increase of novel therapies for rare diseases, among which 50% would have just been impossible to launch for economic reasons [37,38]. The pharma industry contends that shifting those incentives to only rare diseases with a very low prevalence would significantly reduce innovation for all other rare diseases (which represent the biggest number of patients) without bringing the necessary support to ensure the discovery of innovations for those hyper-selected diseases, which require additional specific efforts.

The current arrangements for deploying innovation—rather than innovation itself—are criticised by clinicians who point to delays in reaching the patient with advances in treatment or diagnosis, because of slow approvals, limited and often long-delayed reimbursement, lack of infrastructure, and the failure of guidelines and recommendations to adapt to new possibilities. They cite examples in asthma or lung cancer treatments and diagnosis, such as better tumour biological profiling, advanced invasive and non-invasive diagnostics, immunotherapies and targeted therapies, and highlight inequalities in financing, timely access and availability of newer treatments, particularly in Eastern Europe. For them, the priority in any update to the legislation is to ensure that clinical advances are translated into routine care in a timely and equal manner [39]. 

### 3.2. Unmet Medical Needs (UMNs)

Frequently (and as expressed in some of the EU’s relevant regulation), UMN is regarded as relating to a condition for which there exists no satisfactory method of diagnosis prevention or treatment [40,41]. Rule-of-thumb understandings are that relevant criteria include seriousness of disease, lack of available treatment or of a major therapeutic advantage over existing treatments, and even shortages. However, because there is no universally accepted definition of UMN—some organisations have their own definition for specific purposes—the subject remains wide open to debate, as the workshop testified. WHO has no binding definition, nor does Germany or European Medicines Agency (EMA), and EU rules provide for definitions only in the particular cases of conditional marketing authorisations and some special procedures. Furthermore, UMN is dependent on perspective and context [41].

Attitudes to UMNs accordingly range from insistence on the importance of an agreed definition to flat rejection of the concept of any fixed standard. Some groups favour a definition, although with differing views on what it should consist of, while others tend to be more resistant to a definition. Among patient organizations there are diverse views on a definition and on what it might imply for access. Among HTA bodies there is a desire for clearer guidance on criteria, and some payors have also expressed support for a definition—not least because it could ease their decision-making on what to reimburse. However, among clinicians and drug developers there is perceptible concern over the potential risks of over-rigid definitions imposing arbitrary and ultimately unhelpful limits on care and on innovation [42,43]. 

For those diagnosed with HIV in the years when it was still a terminal condition, the concept had a very clear meaning, and it continues to resonate with those with a life-threatening condition for which there is no clear medical intervention. In Luxembourg, draft legislation is under discussion that would make some provision (on an individual case-by-case basis, through off-label use, compassionate use programmes or temporary authorization) where there is an evident threat to the patient’s health and no effective authorised treatment is available.

Beyond issues of mortality, the debate extends also into how far issues of quality of life can be classified as unmet medical need—where the debate converges with the discussion of incremental innovation [44].

Some cancer patient groups consider the discussion too theoretical, urging a reflection instead on the entire spectrum of care from prevention and early detection to treatment and survivorship, examining every opportunity systematically, and deciding what will have the greatest impact in the light of available resources—which will necessarily vary from country to country [45]. 

The discussion is further complicated if the perspective is shifted from individual needs to a society-wide context. Acceptance of the priority of one patient’s UMN can have implications for the collective—in the case, obviously, of an expensive new treatment (such as for a rare disease) which risks draining the budget available for all patients, potentially leaving them facing an UMN for their existing conditions. UMNs will be numerically greater where many people are affected by a condition, but at the same time it is obvious that UMNs can be more acute in rare conditions even though patient numbers are small [41].

The subject has prompted particular concern because of a link the Commission is discussing—somewhat imprecisely, but nonetheless insistently—between unmet medical need and eligibility for incentives [36]. If, as appears to be the case, the award of incentives is to be governed by the degree of unmet need being met, drug developers express concern over how this modulation is to be practised. A “yes-no” criterion would not correspond to the continuum that is necessarily integral to innovation. At the centre of these reflections are questions over what sort of high-medium-low scale could be put in place, or what complex calculation or algorithm—and how would that relate to the very different priorities of different stakeholder groups. Furthermore, a decision today, on inevitably limited knowledge, would very possibly be invalidated by subsequent findings emerging in light of further evolution of medical science, or the environment, or society. As one cautious panellist observed, “We don’t want to be too restrictive in defining something when science is moving on to places, we cannot maybe imagine today.”

Solutions canvassed for finding greater consensus on UMN included bringing together a wide range of stakeholders to review all the different dimensions of needs in a bid to rank their priority [41].To define UMNs in paediatric rare diseases, for instance, consideration must be given to the multiple needs of children suffering from a disease, of regulatory bodies to make an appropriate benefit risk evaluation, and of HTA bodies to evaluate a new treatment and make recommendations to the payers [36]. Greater reliance on international multi stakeholder collaboration has been functioning in ACCELERATE, a model since 2015 among academia, advocacy, industry, and regulatory agencies to improve and accelerate new drug development for children and adolescents with cancer in what is described as “a patient-centric organisation to solve problems.” The ACCELERATE Paediatric Strategy Forums are run in partnership with the EMA with the Food and Drug Administration (FDA) with the goal to share information in a pre-competitive setting between all stakeholders, to evaluate science on a malignancy or class of compounds, and discuss the landscape of assets in development in the light of defined unmet medical need. This can, it was argued, inform paediatric drug development strategies and subsequent decisions in a way that improves the selection and prioritisation of innovative drugs being evaluated, so that the process is driven by science and by patients’ unmet needs [46].

### 3.3. Access

“It is to be expected,” said one panellist, “that a patient with a life-threatening condition is not indifferent as to whether a potentially life-saving medication is available.” Part of the EU motivation for reviewing the pharmaceutical legislation is a concern that Europe is slow in providing patients with access to treatments—and particularly newer treatments. The link between the discussion of unmet medical need and the discussion of access was also clear from the exchanges at the workshop. As was pointed out, there is powerful evidence of inequality of access across the EU member states to suites of innovative diagnosis or treatment—clear cases of unmet need, and discrepancies that are not necessarily susceptible to remedy simply by an EU agreement on a definition of unmet medical need. 

The WHO perspective is that all member states across its very diverse Europe region suffer problems in access to medicines, with private health expenditure still a major feature (up to 80–90%) in total pharmaceutical expenditure [47]. There is inadequate financing to ensure universal access to essential medicines, inefficiencies in procurement and managing supply chains, insufficient pricing policies and limited negotiating capacity to get lowest prices for quality products, and still substandard quality medicines due to limited regulatory capacity and enforcement. In addition, wide-spread inappropriate prescribing and use are leading to drug resistance and suboptimal health outcomes [48]. 

Even for a wealthy country such as Luxembourg, governing market access means making sure that the drug can be prescribed to the appropriate patients, through registration, pricing and reimbursement. Furthermore, the challenge is still to cope with the many factors that intervene at different levels—the patient, the local level, the national or regional level, and the global level. Smaller member states also have some specific challenges to market access, since they do not always have sufficient capacity and expertise to conduct health technology assessments and rely heavily on evaluations elsewhere. Similarly, a small member state without domestic manufacture depends on other member states to supply the market: Luxembourg relies on Belgium for nearly all its medicines, and Malta relies heavily of the UK—despite Brexit, which has created its own additional challenges—requiring in both cases close collaboration with other national authorities to ensure supply. Such situations also create problems even at the most practical level in terms of the packaging languages.

For Malta, the issue is that the EU single market for medicines does not exist in reality, because of the fragmentation practised by drug companies who choose where to market—and where not to market—their products. Smaller or more remote or poorer countries are disadvantaged in this calculus, they argue. They see drug firms as benefiting from a freedom that countries are denied [49]. 

The EU regulatory framework, introduced in the last century and last updated in 2004, was designed to provide safe, efficacious, and high-quality medicines to patients in the EU [34,40]. The new ambition to use the regulatory framework to tackle access issues or to “ensure sustainability of the member states’ public health systems”—as the Commission proposes—is a novel concept in the EU legislative arena. It has generated concerns, particularly in industry, since access is a member state rather than an EU-level responsibility. There are questions over whether changes to the overarching regulatory framework will have any positive effect, or even lead to unintended further delays in EU patients’ access to innovative medicines, if the effect is to accelerate EU decline in innovation.

The Commission is promoting reflection on whether and how to link rewards for innovation with conditionalities that can address this aspect of access. It is exploring how to encourage—or pressure—companies to launch their centrally authorised products across the entire EU as a contribution to remedying difficulties in access. Companies are not opposed to filing in the 27 Member States, but they have pointed out that many local conditions often militate against this. Procedural burdens exist after filing: for example, some local procedures cannot start before a certain number of countries have already started to process applications for approval of the medicine. Furthermore, the heterogenous required data package among Member States can make it difficult to fulfil all requests of all Member States, notably when standards of care differ, as well as the willingness and ability to pay. Companies therefore respond with questions about how far obligations would be reciprocal: in other words, if companies are obliged to market their products, are member states similarly obliged to fund the access. The concept of obligatory pan-EU marketing also raises familiar and much-rehearsed questions about compatibility with external reference pricing, and the confidential discounting systems that have grown up around it [50]. Other mechanisms advanced as possible contributions to easing access include enhancing competition from off-patent products, by facilitating faster entry of generics and biosimilars, and repurposing [51].

Patients will naturally request access to the best quality of care at the individual level. Access for breast cancer care is improving for European women, with current screening, diagnosis and treatment—as well as cost coverage—fairly good, but there is still a lot of inequality in access to new therapies, and coverage is less good for genetic and genomic testing [52]. Most specialist breast units still operate without certifications, and there are national gaps in specialist breast nurse and data managers and in practical advice, support and counselling from appropriate specialists [53].

Doctors too will have their own perspective, seeking maximum liberty of prescription and treatment. However, payers, with a duty to the collective, will also advocate in favour of their own broader interests to make best use of public resources, with some of them already advancing the notion of a new social contract with pharmaceutical companies. It is not possible, it is recognised, for everybody to be given access to everything, and the challenge of scarce resources will always be there, along with the need for some decisions on prioritisation. However, some apprehensive patients raise the risk that if new criteria are to be created for access, medications currently marketed might no longer be available, depriving existing patients, and they are insisting on the importance of the patient voice being heard in all this.

Major reasons for unequal access to novel drugs include differences in budgets (notably with national variations in health care spending), differences in timing of drug reimbursement decisions, differences in organization of healthcare, differences in pharma priorities—often the real challenge for a company to file in 27 Member States at the same time, notably for SMEs who have by definition limited resources—and differences in access to clinical trials locally and across borders [54]. (There is wide diversity in use of medicines between and within groups of countries, and by group of medicine (e.g., large differences in uptake of immunotherapy medicines even within similar countries, and very low uptake in poorer countries).

Access to medicines and health products is part of the WHO European Programme of Work 2020–2025, and approaches include convening stakeholders (including patients, non-state actors and the pharmaceutical industry) to work towards a new social contract through high level dialogue that could better identify and support the correction of vulnerabilities in regulatory, production, procurement and supply chains [55]. The Oslo Medicines Initiative, led by the WHO Regional Office for Europe and the Norwegian Ministry of Health and Care Services and the Norwegian Medicines Agency, is expected to deliver its final recommendations in 2022 on equitable and sustainable access to effective, innovative and affordable medicines. Furthermore, a World Health Assembly resolution is awaiting implementation on improved market transparency, strengthening information systems and expanding voluntary intercountry collaborative platforms and procurement.

### 3.4. Security of Supply

The COVID pandemic gave new prominence to the persistent problem of shortages of medicines, reinforcing concerns over vulnerabilities in Europe’s pharmaceutical supply chain [56]. The European Commission [10] frankly admits that shortages are a growing problem, and one that will not solve itself, as its recent report concluded. That conclusion was echoed in another recent report from Organisation for Economic Co-operation and Development (OECD), presented by one of the panellists. Unsurprisingly, the challenges were the focus of much of the comment by panellists—not least on behalf of patients, who stand to suffer the most from shortages. Notably, the EU’s Cancer Mission has also taken account of the risks that drug shortages pose to its aims: even a brief shortage is a major complication for patients.

Repeated testimony was offered in the discussions of complicating factors: the timing of reports of shortages varies across countries, as does the threshold for reporting, and there are problems arising from poor data quality. The broad consensus among panellists was that there is a need for a common European response to this problem. Many of the remedies urged by panellists highlighted the importance of a multi-stakeholder approach, involving healthcare professionals, pharmacist organisations, wholesale distributors, manufacturers, patient representative groups, health authorities and marketing authorisation holders. Because the chain has multiple parts—any one of which can generate shortages at least on a temporary basis—it is logical to seek the necessary multiple different solutions through a multi stakeholder approach. However, the essence is coordination, since there are limits to what each different group can achieve on its own, and comprehensive solutions can emerge only when stakeholders work together.

Solutions could emerge best from clear diagnosis of the problem, it was argued: systematic information was needed on who was the first to report a shortage, and when it was notified. Other key areas of inquiry should be what therapeutic areas are affected, whether the direct cause was internal to the product (such as regulatory or quality or withdrawal or other product-related issue) or external (such as shipping delays, unexpected demand, or stockpiling). However, monitoring and mitigation can do only so much in the absence of more fundamental change. 

Better communication was urged as essential, between and among manufacturers of ingredients and finished products, parallel traders, wholesale distributors, and pharmacies. One suggestion was for the creation of a common taxonomy of shortages. Greater harmonisation of national monitoring systems would allow more robust comparisons within and across countries, and potentially better understanding of the impact on patients and the underlying root causes. 

It was stressed that the issue cannot be viewed in isolation from broader health policy issues, including pricing and reimbursement. Reimbursement regimes that impose very low prices may trigger commercial decisions to withdraw or even cease manufacture of some products where the economics are unsustainable, putting supply at risk. This risk appears all the greater in the case of the products already disproportionately prone to shortages—low-priced off-patent products. Increasing use of tendering mechanisms that focus on price are accelerating the risk of dependence and shortages, and it is even more true when tenders define one single winner.

Weaknesses in sourcing and supplying medicines, and the associated gaps in information and monitoring of supply chains, predispose another problem, according to WHO, which recognises an inability to halt falsified medicines in its Europe region [57].

More broadly, the meeting reflected on the global dimension of the challenge—all the more so as the conflict in Ukraine illustrated both the fragility of international supply chains and the grave implications for patients when there is wide-scale disruption of their medication. Potential solutions included shifts in manufacturing arrangements, to avoid particular components being highly concentrated at a limited number of manufacturing sites—although this has to be seen in the broader context of the economics of manufacturing. The vision set out by some panellists was of a global approach engaging all relevant actors, in the conviction that any solution is going to be multinational and not just national. Urgency too was emphasised, since even when good principles and guidelines are generated and accepted, it can take a long time before they are translated into concrete actions.

### 3.5. Adapting to Progress

The concern was evident among many panellists about Europe’s global competitiveness and its capacity to exploit emerging opportunities in drug development. Formalizing existing pathways for innovative medicines and ensuring expedited assessment pathways was the focus of much comment from drug developers, with an emphasis on aligning Europe’s drug assessment procedures and regulatory approvals more closely with the evolution of science.

To support and accelerate product development and authorisation, particularly in areas of unmet need, there were calls for standardised research protocols and common platforms for rapid recruitment into adaptive clinical trials. The ‘eternal’ trials like Remap-cap and Recovery that have been adapted for COVID-19 should, it was argued, be utilised for other areas such as antimicrobial medicines, maximising the ability shown in the pandemic to achieve remarkable results in terms of developing vaccines in 10 months rather than 10 years [58,59]. Regulators must be allowed flexibility to adapt procedures to the latest science whenever needed and not only in times of pandemic, it was argued.

There were arguments in favour of more acceptance of real-world data (RWD), in light of the European Centre for Disease Prevention and Control (ECDC’s) demonstration during the pandemic of how important RWD are to making decisions, and as a complement and a useful addition to clinical trial data. However, there will have to be better integration of RWD, through systems able to speak to each other, so that they can play a role in the approval system of new medicines [60]. Definitions will be needed as to how far RWD and real-world evidence (RWE) are acceptable, and for which purposes—market access, reimbursement, registries, pragmatic trials, etc. Acceptability may also depend on how data are collected or curated, on stakeholders’ involvement, on digitalization and management, and interoperability of results [61].

Unflattering trans-Atlantic comparisons were offered of the relative speed and scope of fast-track expedited assessment approaches to drug approval. The US FDA’s breakthrough designation procedure had processed many more products, and much faster, than EMA’s approximate equivalent scheme for Priority Medicines (PRIME). A higher proportion of products through the US scheme had also obtained marketing approval than through PRIME. The contrast was likely to persist as long as PRIME remained as nothing more than an optional scheme, it was argued. Instead, the scheme should be elevated to formal status by legislation that places it on a similar footing to the EU’s established authorisation procedures. It should be opened up to cater for new indications for approved medicines, and should be adequately resourced [62] (The issue of resources for regulators recurred during the meeting, and is also dealt with below under “Efficiencies”).

Similarly, there is an appetite for generalisation in Europe of the rolling reviews introduced during the COVID pandemic as a way of speeding the assessment of vaccines and other relevant therapeutics. The argument is that if the demonstrable time-savings are a valid approach in emergencies, there is good reason for providing for their use for innovative therapies in more routine settings, since the urgency of assessment is as great for patients who stand to benefit from innovation. Of course, if these time savings are to translate into routine regulatory reviews- resources need to be increased dramatically

There were calls for greater synergy between the medical devices’ legislation and the pharmaceutical legislation, in view of the increased significance and evident potential of combination products. 

Traditionally, tumours have been diagnosed, classified, and treated based on their site of origin (e.g., brain, lung, breast, prostate, or thyroid) and further histologically subtyped (e.g., small cell vs non-small cell and adenocarcinoma versus squamous cell cancer) [63,64]. However, with the arrival of powerful NGS technologies permitting rapid, accurate, and inexpensive interrogation of clinical samples, new observations suggest an expanded or different approach to determining cancer nosology, one driven by the unique molecular features of the tumour [65]. 

An abundance of data now indicate that molecular aberrations may be shared across multiple tumours with distinct sites of origin. This has led to tissue agnostic drug development and approvals [65,66].

Precision oncology’s raison-d’être is to offer “the right drug for the right patient at the right time” a process enabled by transformative tissue and blood-based genomic technologies [67]. With the landmark approvals of pembrolizumab for the treatment of patients whose tumours have high microsatellite instability or high-tumour mutational burden (TMB ≥ 10) and larotrectinib and entrectinib for those harbouring NTRK fusions, a regulatory pathway has been created to facilitate the approval of histology-agnostic indications. Near future potential tissue agnostic targets include RET, KRAS G12C, and NRG1 [68]. While success has been seen in targeting the abovementioned alterations, other tissue agnostic trials for TSC1/2 and CDK alterations have been negative. In addition, BRAF V600 presents a unique target and critical lessons can be learned from histology dependent and histology-independent trials using BRAF inhibitors [69,70].

Histology-agnostic development may not be a feasible approach for all future oncology drugs [68]. Future efforts in histology-agnostic drug development should ensure that patients receive therapeutic agents targeting a true driver mutation and acknowledge co-occurring alterations in known resistance pathways [68,71]. The evaluation of potential tissue agnostic treatments in oncology raises a number of issues for consideration of orphan drug designation such as what factors should be considered in defining a tissue agnostic disease or condition at the time of request for orphan drug designation, how FDA and EMA should consider orphan drug designation requests of the same product for a tissue agnostic disease or condition and also for a disease based on traditional disease nomenclature [72]. 

Future approvals should mirror the tumour landscape and the efficacy results of the approvals to date, in trials designed with patient selection criteria and statistical analyses suitable for the analysis of multiple heterogeneous histologies [68].

### 3.6. Efficiency

The starting point for discussions of health policy in Europe is often that there is no such thing as a European health system, which means that health policy is inevitably a series of compromises between the national and European level, raising repeated questions over efficiency [73]. Nonetheless, without going so far as to seek radical change in responsibilities between member states and the EU, a range of options were discussed by panellists for improving efficiency.

Digitisation is seen as a major opportunity, linked to the technical adaptations such as remote consultation and monitoring given new impetus by the pandemic, and the prospects for more effective capture and use of interoperable health data [74]. Sharing of data at the EU level was depicted as key to fostering innovation, to acquiring a comprehensive view of medical needs and availability of medicines across the EU.

There is scope, it is widely acknowledged, for improvements in healthcare organisation that can promote innovation, fulfil unmet needs, reduce inequalities in access, and secure supply better. Over and above increases in budgets—and any increases in funding must be carefully targeted, it was emphasized—there are optimisations that can be achieved in drug approval processes and HTA, at national and at EU level. Closer alignment of the inevitably distinct functions of regulatory and HTA processes could limit the need for developing and submitting the same data twice, or avoidable inconsistencies in requirements. Increased opportunities for early dialogue—between companies and authorities, but also with inputs from patients and other stakeholders—on products in development could help to establish at the very least what levels of uncertainty could be acceptable and how they are going to be measured. Regulators could consequently streamline any redundant bureaucratic processes at EU or national level. 

Adaptations made to accelerate drug development during the COVID pandemic showed where administrative delays in the processes could be eliminated without risks to safety, and studies suggest that extending these adaptations more widely could save patients’ lives. An illustrative analysis of 11 recently authorized oncology treatments shows that the regulatory steps between Committee for Medicinal Products for Human Use (CHMP)’s opinion and EC decision together accounted for 18,600 years of potential life lost, although the full extent of life years lost is far greater when considering all oncology indications [75].

Access to clinical trials can be improved by organisation of dedicated clinical trial units in hospitals and by dedicated provision for participation by patients across borders, overcoming current barriers of informed consent and insurance. There are also gaps in the current cross-border legislation that could maximise synergies. The review of EU screening guidelines is seen as a potential aid to early diagnosis and could also stimulate the uptake of and support for molecular diagnostics, with better frameworks for lab infrastructure, molecular tumour boards, and staff training. 

Better understanding of the consequences of policy decisions or therapeutic choices could permit significant improvements. Development of databases on resource use and outcomes would allow comparative studies of efficiency in different countries/regions/populations. Studies could reveal to what extent countries spend on cost-effective medicines—on which there is currently little evidence, because it is not investigated. Similarly, there is little information on waste in health care spending—and the lack of information means there are few incentives for reducing it. New options for prevention, diagnosis, and treatment are useful only if there are also data on how they should be implemented in the health care system. The new evidence landscape offers more alternatives and more choices, and creates an increased need for evaluation.

## 4. Conclusions

In this vision, the necessary collection of data would be conducted not just for the purposes of administration but also to feed into changing management of systems for the better. The need is for integrated approaches that look at the big picture beyond everyone’s own comfort zones. It is evident from recent experience that increased resilience and flexibility is essential in health systems. As one panellist expressed it: “You have to zoom out and look at your problem from a level above—improving systems in order to deliver best patient care will be the challenge”.

## Data Availability

Not applicable.
